# Proteasome β5 subunit overexpression improves proteostasis during aging and extends lifespan in *Drosophila melanogaster*

**DOI:** 10.1038/s41598-019-39508-4

**Published:** 2019-02-28

**Authors:** Nga N. Nguyen, Anil Rana, Camille Goldman, Rhiannon Moore, Justin Tai, Yongchan Hong, Jingyi Shen, David W. Walker, Jae H. Hur

**Affiliations:** 1 Department of Biology, Harvey Mudd College, Claremont, CA 91711 USA; 2 Department of Integrative Biology and Physiology, University of California, Los Angeles, CA 90095 USA; 3 Department of Biology, Pomona College, Claremont, CA 91711 USA; 4 W. M. Keck Science Department, Pitzer College, Claremont, CA 91711 USA; 5 Molecular Biology Institute, University of California, Los Angeles, CA 90095 USA

## Abstract

The β5 subunit of the proteasome has been shown in worms and in human cell lines to be regulatory. In these models, β5 overexpression results in upregulation of the entire proteasome complex which is sufficient to increase proteotoxic stress resistance, improve metabolic parameters, and increase longevity. However, fundamental questions remain unanswered, including the temporal requirements for β5 overexpression and whether β5 overexpression can extend lifespan in other species. To determine if adult-only overexpression of the β5 subunit can increase proteasome activity in a different model, we characterized phenotypes associated with β5 overexpression in *Drosophila melanogaster* adults. We find that adult-only overexpression of the β5 subunit does not result in transcriptional upregulation of the other subunits of the proteasome as they do in nematodes and human cell culture. Despite this lack of a regulatory role, boosting β5 expression increases the chymotrypsin-like activity associated with the proteasome, reduces both the size and number of ubiquitinated protein aggregates in aged flies, and increases longevity. Surprisingly, these phenotypes were not associated with increased resistance to acute proteotoxic insults or improved metabolic parameters.

## Introduction

Aging is characterized by an increase in accumulation of cellular damage over time that leads to heightened susceptibility to both intrinsic and extrinsic causes of mortality^1^. Though the effects of aging are widespread, many specific hallmarks have been identified that may be causal to the aging process^2^. Among these, we focus on the role of protein damage and loss of protein homeostasis (“proteostasis”) as a source of many of the detrimental effects of aging^3^.

Highly reactive molecules, in particular, reactive oxygen species (ROS), are generated as a byproduct of metabolic processes that are required to generate energy for life processes^4^. These often react in uncontrolled manners with cell components, producing unwanted oxidation products. In particular, as a major constituent of cells, proteins bear much of this oxidative damage load, and the reaction of free radicals with proteins can result in the alteration or loss of amino acids, negatively affecting protein folding and function^5^. Thus, metabolic processes present an unavoidable source of damage for cellular components, including proteins^6^. Oxidative damage accrued during the aging process is particularly detrimental to components of proteostatic pathways as loss of efficiency of these pathways can result in acceleration of rate of damage accrual by all cell components, including further damage to proteostatic pathways^7–9^. Oxidized and aggregated proteins are particularly effective at binding and inactivating proteasomes which degrade damaged proteins^10–12^. This can result in a vicious cycle of increased damage that may result in increased acceleration of aging phenotypes.

Two major pathways, autophagy and the ubiquitin proteasome system (UPS), function to remove damaged proteins by degradation to make room for new syntheses^13^. The autophagy pathway involves the packaging of whole volumes of cells into specialized vesicles, the autophagosomes, and their subsequent digestion through the lysosome degradation pathway^14^. Reductions of autophagy pathway component functions have been implicated in increased susceptibility to diseases characterized by a reduced ability to degrade and recycle cellular components such as mitochondria^15^, and the activity of the autophagy pathway has been shown to decline with aging which may lead to the exacerbation of aging phenotypes^16,17^. Moreover, transgenic studies have shown that increasing activity of the autophagy pathway can increase stress resistance and longevity in multiple model organisms^18–20^.

In contrast to the autophagy pathway which is associated with the removal and processing of cellular volumes *en masse*, proteins and protein complexes are degraded individually in the cytoplasm by specifically targeted proteases, the major one being the proteasome which functions as a part of the UPS^21^. Here, ubiquitin transferases and ligases target specific proteins for degradation by the attachment of long ubiquitin chains that are linked by K48 residues. Proteins thus tagged with at least 4 K48-linked ubiquitins are recognized and subsequently degraded into small polypeptide fragments by the proteasome^22^. The proteasome is a large proteolytic complex that consists of a barrel-shaped 20S core particle (CP) capped by 19S regulatory particles (RPs) at one or both ends to form the 26S and 30S assemblies, respectively^23^. While both the CP and RP in isolation have been shown to house critical activities, the 26S assembly (CP + RP) and the 30S assemblies (RP + CP + RP) are thought to provide the great majority of the proteolytic activity in the UPS^24^. Moreover, the activities of the 20S can be distinguished from the activities of the 26S and 30S assemblies by the requirement for ATP by the 26S and 30S assemblies.

The CP houses the proteolytic functions and the RPs allow for selective, ATP dependent entry of ubiquitinated substrates into the CP for degradation. The canonical CP consists of 28 subunits, two each of 7 different α and 7 different β subunits. These are organized as four heptameric rings, two identical α subunit rings comprised of α1-α7 subunits and two identical β subunits rings comprised of the β1-β7 subunits. These rings associate in an αββα conformation to form a barrel like structure with proteolytic activities housed inside the chamber. Three β subunits are associated with the three proteolytic activities of the proteasome: β1 (caspase-like), β2 (trypsin-like), and β5 (chymotrypsin-like). Other assemblies that involve other subunits and different stoichiometries are known, including the well characterized immunoproteasome^25^, testis-specific proteasomes^26^, thymoproteasomes^27^, and an evolutionarily conserved CP that incorporates additional α4 subunits in place of the α3 subunits^28,29^.

As with autophagy pathway components, upregulation of parts of the UPS has been shown to be effective at extending lifespan across multiple models^30,31^, and increases in aging-associated phenotypes has been shown to be associated with loss of UPS activity in multiple models^32,33^. In particular, overexpression of a ubiquitin ligase was shown to be sufficient to reduce protein aggregates, confer resistance to proteotoxic stress, and extend longevity in *Drosophila*^34^. Similarly, overexpression of specific regulatory subunits in the cap or core of the proteasome have been shown to be sufficient to increase activity of entire 26S and/or 30S canonical proteasomes, and this increased activity has been shown to be effective at mitigating the effects of proteotoxic elements and extending lifespan^35–38^. Among the CP components, the β5 subunit, in addition to being the location of the chymotrypsin-like proteolytic activity of the proteasome, has been shown to have a regulatory function in *C. elegans* and human fibroblasts. In these models, continuous overexpression of β5 was shown to be sufficient to induce the expression and activation of all other subunits of the proteasome, including those involved in the CP and RP. The resulting increases in 26S and/or 30S assemblies were associated with significant elevation of all of the proteasome associated proteolytic activities^38,39^. Consistently, depletion of the β5-associated chymotrypsin-like activity in mice has been shown to result in multiple detrimental phenotypes including shortened lifespan, decreased body weight and altered metabolism, muscle wasting, and accumulation of polyubiquitinated peptides^40^.

Organismal effects of the overexpression of this critical subunit has thus far been limited to *C. elegans* and the long-term effects of β5 overexpression during adult stages only have not been reported. Accordingly, we characterized the biochemical and physiological effects of overexpressing the fruit fly homolog of the β5 subunit of the proteasome (CG12323) in *D. melanogaster* only during adulthood. We find that in flies, β5 does not appear to have a regulatory role for other proteasome subunits, and its overexpression does not cause an increase in transcription of all other subunits of the proteasome. Its overexpression is nevertheless sufficient to increase the β5-associated chymotrypsin-like activity of the 26S/30S proteasome in *in vitro* assays of proteasome activity. Moreover, we find that increasing the chymotrypsin-like activity of the 26S/30S proteasome during adulthood in flies does not alter multiple parameters reported to be associated with chymotrypsin depletion in mice, including body weight, metabolism, and muscular function. We find that increased chymotrypsin-like activity is, however, sufficient to reduce the presence and size of ubiquitinated protein aggregates and to extend lifespan, suggesting that β5 subunit expression and chymotrypsin-like activity are limiting for longevity in flies.

## Materials and Methods

### Fly genetics and culture

UAS-β5 (UAS-CG12323) flies were generated by phiC31 integrase mediated transformation of a pUASTattB plasmid^41^ carrying *D. melanogaster* proteasome subunit β5 cDNA (Drosophila Genomics Resource Center, NIH 2P40OD010949) into a previously described insertion line (*attp33*)^42^ using standard methods. The *daughterless-GAL4* driver and *w*^*1118*^ genetic background lines were obtained from the Bloomington *Drosophila* Stock Center (NIH P40OD018537), and the *daGS* driver was obtained from the original creators^43^. All flies were cultured on standard agar-cornmeal-yeast-sugar media^44^ in humidified incubators at 25 °C, on 12:12 hour light:dark cycles.

### Quantitative Real-Time Polymerase Chain Reaction

Total RNA from adult flies was extracted using TRIzol reagent (ThermoFisher Scientific, Waltham, Massachusetts, USA) following manufacturer protocols. RNA preparations were treated with DNAse I (ThermoFisher) to remove genomic contaminants and reverse-transcribed into cDNA following manufacturer protocols (RevertAid, ThermoFisher). cDNA amplification during a 50-cycle PCR (Power SYBR Green Master Mix, ThermoFisher) was monitored using a Mastercycler Realplex 2 real-time PCR machine (Eppendorf, Hamburg, Germany). Amplification of *actin5C*, *actin42a*, *α-tubulin*, *RpL32*, or *eEF1α2* was used to normalize loading among samples. Primer sequences for *actin42a*, *α-tubulin*, *RpL32*, and *eEF1α2* were previously reported in an analysis of qPCR reference genes^45^. Other primer sequences are: *actin5C*: 5′-TTGTCTGGGCAAGAGGATCAG-3′ and 5′-ACCACTCGCACTTGCACTTTC-3′, *β5*: 5′-AACTTCGATCACGGCACCAC-3′ and 5′-GGGAGCCAATGTACGATCCA-3′, *β1*: 5′- GTGGTCATTGGAGCCGATTC-3′ and 5′- TGCGGCAGCAGTACACTTTG-3′, *β2*: 5′-CGCAATGCAACTCTTTTGAA-3′ and 5′-GTAATGGATCTTGGCGCAGT-3′, *α2*: 5′-GAGCAGCACAGTGTACATCG-3′ and 5′-CAGCTGTGACACTGGAATCG-3′, *α3*: 5′-GCGTTACCAGTTCAGCTACG-3′ and 5′-GCCGTACTTGTTGTCCCATC-3′, *rpn10*: 5′-GATCTGGACCTGGAAACGAA-3′ and 5′-TGGACATCTGCATAGCGAAG-3′, *rpn13*: 5′-ATTTTGTGCGTGCTCTGGAG-3′ and 5′-CCACTGAAGTTGGGGTTTCG-3′, *rpt2*: 5′-ATCATGGCCACCAATCGTAT-3′ and 5′-TTCGCTAAGGTTCACGTCCT-3′, *rpt3*: 5′-ATCGCTACATCGTTTTGGCC-3′ and 5′-GGCGGGAGTAGTGTGTATGA-3′, *rpn6*: 5′-TTGAGTGGGCCAAACAGGAG-3′ and 5′-AGGCCTCCGTGTACAAAGCA-3′, *rpn11*: 5′-TTTGTTGCTGCTTTCGACGA-3′ and 5′-ACCTCCAAGACGTAGCAGAC-3′.

### 26S/30S Proteasome chymotrypsin-like activity assay

Cell lysates were prepared from fly thoraces and heads by homogenization in assay buffer (50 mM Tris-HCl pH 7.5, 5 mM MgCl2, 1 mM DTT) and incubated at room temperature for 30 min with either 40 µM proteasome inhibitor (PSI)^46^ or carrier (DMSO). Enzyme activity was initiated by supplementing with 5 mM of a fluorogenic substrate specific for chymotrypsin-like activity (Suc-LLVY-AMC) and 1 mM ATP in assay buffer to measure 26S or 30S activities which comprise the bulk of the proteolytic activities of the proteasome in UPS^24^. Fluorescence of the liberated AMC reporter was measured as an increase over time of emission at 480 nm due to excitation at 380 nm, using a microplate reader (SynergyHTX, BioTek, Winooski, Vermont, USA). In order to distinguish proteolytic activity provided by the proteasome from any background proteolytic activities, each sample was measured twice in parallel, in the presence of the proteasome inhibitor (PSI) and in the presence of carrier only (DMSO), and the difference in proteolytic activity was recorded as proteolytic activity specific to the proteasome. All samples were normalized by total protein content, as measured by the Micro BCA Protein Assay kit (ThermoFisher Scientific) following manufacturer instructions.

### Survivorship assays

All flies were developed through eclosion on standard agar-cornmeal-yeast-sugar media^44^ then collected within 2 days of eclosion onto media supplemented with 10 µg/ml RU486 and 0.1% ethanol (drugged) or 0.1% ethanol only (control). Flies were allowed to mate over 2 days, sorted on light N_2_ anesthesia into groups of 30 females, and continuously maintained from then on in vials containing 2 ml drugged or control media. Flies were kept in a humidified, 25 °C chamber with a 12:12 light:dark cycle and switched to new vials every 2–3 days. For lifespan assays, each vial of flies was scored for death every 2–3 days. For hyperoxia resistance assays, flies were aged identically to the lifespan assays and then maintained in a humidified chamber at room temperature at >92% O_2_ and scored for death twice a day during the assay period. For heat stress resistance assays, flies were aged identically to the lifespan assays and then kept in a humidified 37 °C incubator and scored for death every 2 hours.

### Capillary Feeding (CAFE)

Feeding was monitored as previously described^47^ in groups of 10 flies per vial for approximately 8 hours in a humidified, 25 °C chamber.

### Weight

Flies were weighed on an analytical balance in groups of 10 in tared 2 ml microcentrifuge tubes.

### Respiration

Relative CO_2_ production was measured as previously described^48^. Briefly, air-tight respirometers were constructed by fitting 50 μL capillaries into 1 ml pipette tips which contained a gas permeable chamber filled with soda lime. Each respirometer was used to monitor respiration from 5 flies over 2 hours, after a 15 minute acclimation period, in a humidified 25 °C incubator by allowing the respirometer to draw up a colored liquid against gravity. Flies consumed the available O_2_ in the respirometers to produce CO_2_ which was, sequestered by the soda lime, causing the negative pressure to draw up the colored liquid against gravity. Every assay contained at least four atmospheric controls (respirometers without flies) to control for any changes due to changes in atmospheric pressure during the assay period.

### Climbing

Climbing ability was assayed in groups of 15 flies in 30 cm climbing vials that had been marked at 10 cm intervals. Flies were acclimated for at least 1 hour (with food) prior to testing, at which point the food vial was disconnected from the climbing vials. The position of the flies 20 seconds after being tapped down to the bottom was recorded and scored based on which segment they climbed to (bottom = 1, middle = 2, top = 3). Each vial was tested 5 times with 10 minutes of rest between tests and these were averaged as technical replicates.

### Immunostaining and image analyses

Flies were fixed for 20 min with 3.7% formaldehyde in PBS, and after fixation, hemi-thoraces were dissected. Samples were rinsed three times with 0.2% Triton X-100 in PBS (PBST) and blocked for 1 h at room temperature in 3% BSA in PBST. Primary antibodies (anti-FK2, mono- and polyubiquitinylated conjugates monoclonal antibody, BML-PW8810, Enzo) were diluted 1:250 in 5% BSA in PBST and samples were incubated overnight at 4 °C. Samples were rinsed thrice with PBST and incubated in a mix of 1:250 diluted secondary antibodies (anti-mouse AlexaFluor-488, Invitrogen) and a stain (phalloidin AlexaFlour-568, Invitrogen) in 5% BSA in PBST for 3 hours at room temperature. Samples were rinsed three times with PBST and mounted in Vectashield mounting medium (Vector Labs) and imaged using a Zeiss single point LSM 5 exciter confocal microscope. Protein aggregate and total muscle areas were quantified using ImageJ using the analyze particle features and the aggregate values were normalized by the area of muscle.

### Statistical analyses

All statistical tests were performed using Microsoft Excel, the R statistical package^49^, or Graphpad Prism (version 5.03, GraphPad Software, La Jolla California USA, www.graphpad.com) with significance cutoff at (*)p < 0.05, (**)p < 0.01 and (***)p < 0.001.

## Results

### β5 subunit overexpression does not transcriptionally upregulate other proteasome subunits

**Figure 1:**
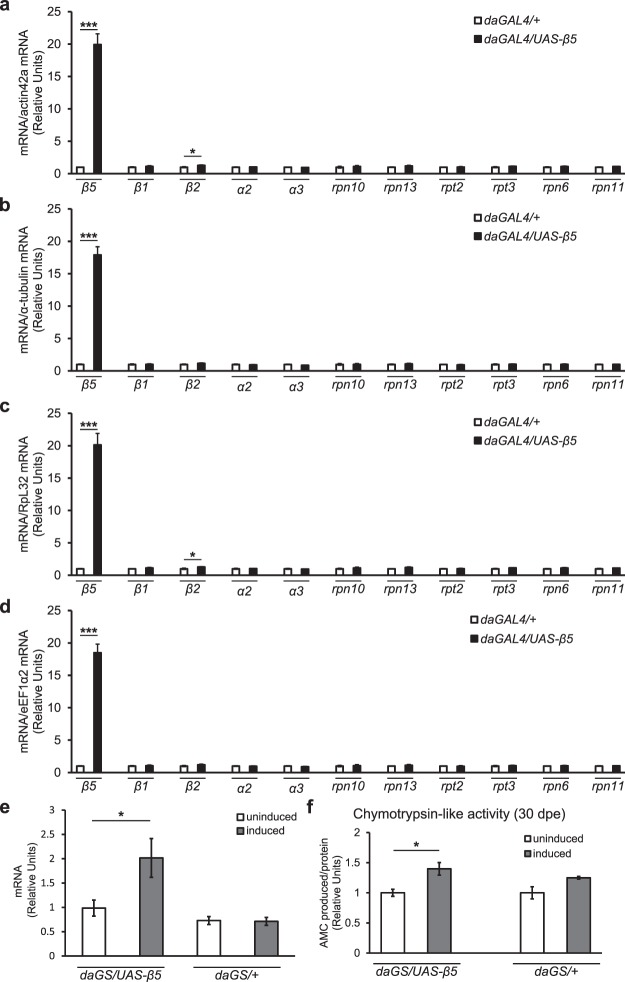
Overexpression of the β5 subunit of the proteasome causes increased chymotrypsin-like activity without consistent transcriptional upregulation of other proteasome subunits. Strong, ubiquitous, and constitutive overexpression of the proteasome β5 subunit in mated female flies at 10 days post eclosion (dpe) does not result in consistently altered transcript levels of different subunits of the proteasome core (*β1*, *β2*, *α2*, *α3*) or cap (*rpn10*, *rpn13*, *rpt2*, *rpt3*, *rpn6*, *rpn11*) when normalized to four different reference genes: (**a**) *actin42a* [CG12051], (**b**) *α-tubulin* [CG1913], (**c**) *RpL32* [CG7939], and (**d**) *eEF1α2* [CG1873]. 5 replicates, 5 flies per replicate. *p < 0.05, ***p < 0.001, t-test. (**e**) Overexpression of the β5 subunit in mated female flies using a drug-inducible driver produces a moderate increase in β5 transcript level at 10 dpe. Expression of the β5 subunit is not influenced by the presence of the inducing drug (RU486/mifepristone) in driver-only controls. At least 9 replicates, 3 flies per replicate. *p < 0.05, t-test. (**f**) Induction of β5 subunit overexpression during adulthood in mated female flies results in a significant elevation in 26S and/or 30S proteasome specific chymotrypsin-like activity by 30 dpe. 5 replicates, 5 heads and thoraces per replicate. *p < 0.05, t-test. All error bars represent standard error.

Previous reports aimed at increasing the activity of the proteasome had shown that the β5 subunit is regulatory in human cell cultures and *C. elegans*, and in these contexts, expression of the β5 subunit is sufficient to induce increased transcript levels and protein subunits of the entire proteasome^38,39^. In order to determine if β5 expression is also regulatory in fruit flies, we over-expressed the *D. melanogaster* homolog of the proteasome β5 subunit (CG12323) by constitutively driving robust, ubiquitous expression of a cDNA construct using the GAL4/UAS expression system with a daughterless promoter^50^. In order to assess the effects of β5 overexpression, we specifically checked the transcript levels of 10 other subunits of the proteasome chosen based on their importance to proteasome function and previous evidence for their role in proteasome regulation. Specifically, along with β5, we checked the transcript levels of the remaining two proteolytic subunits (*β1* and *β2*)^23^, two of the three α subunits involved in gating the CP (*α2* and *α3*)^51^, both RP subunits involved in ubiquitinated substrate recognition and gating (*rpn10* and *rpn13*)^52,53^, two RP subunits involved in the ATPase activity of the RP (*rpt2* and *rpt3*)^54,55^, and two RP subunits which had previously been reported to be regulatory in *D. melanogaster* (*rpn11*)^35^ and *C. elegans* (*rpn6*)^37^. In addition, previous reports have shown that the choice of reference genes in quantitative real-time PCR analyses can result in misleading results. Accordingly, in order to assess the transcript level effects of β5 overexpression, we normalized each result to four different reference genes which were previously reported to be among the most stable^45^. In every case, our over-expression scheme resulted in robust overexpression of the β5 subunit (approximately 20-fold increase for all four reference genes, Fig. 1a–d). Only one of the 10 subunits tested (*β2*) was found to be slightly overexpressed (~30%) when the qRT-PCR data was normalized to two of the four different reference genes tested (Fig. 1a,c, see Supplementary Table S1). Therefore, we find that despite approximately 20-fold overexpression of the β5 mRNA relative to a genetic background control, there were no across-the-board significant and consistent differences in transcript levels of other subunits in the CP or the RP in adult flies.

### β5 overexpression results in increased chymotrypsin-like activity of the proteasome

To reduce the likelihood that genetic background effects might interfere with subsequent experimental outcomes, for the rest of the assays, the UAS-β5 transgene was induced using a drug-inducible *daughterless-GeneSwitch* driver (*daGS*)^43^. Accordingly, for this and all subsequent assays, comparisons were only made between genetically identical siblings that were either given the inducing drug (RU486/mifepristone) or the carrier (ethanol) only. Any effects of the inducing drug itself was controlled using the progeny of the cross between the genetic background used to construct the UAS-β5 transgenic line (*attp33*)^42^ and the *daGS* driver. qRT-PCR analyses revealed that the *daGS* driver induced a more moderate overexpression of β5 under our “induced” conditions (approximately 2-fold, Fig. 1e). Moreover, *in vitro* enzyme activity assays using a fluorogenic substrate specific to the chymotrypsin-like activity of the proteasome and a proteasome-specific inhibitor (PSI) revealed a significant increase in the β5-associated chymotrypsin-like activity 30 days post eclosion (dpe) (Fig. 1f) upon induction of β5 overexpression during adulthood, showing that β5 overexpression is sufficient to increase proteasome chymotrypsin-like activity in flies. We did not see a significant difference in chymotrypsin-like activity at 10 dpe or 45 dpe (see Supplementary Fig. S1), which suggests that β5 transcript levels are a limiting factor for proteasomal chymotrypsin-like activity only during a specific period between 10 dpe and 45 dpe.

### Boosting chymotrypsin-like activity in adults extends lifespan

**Figure 2:**
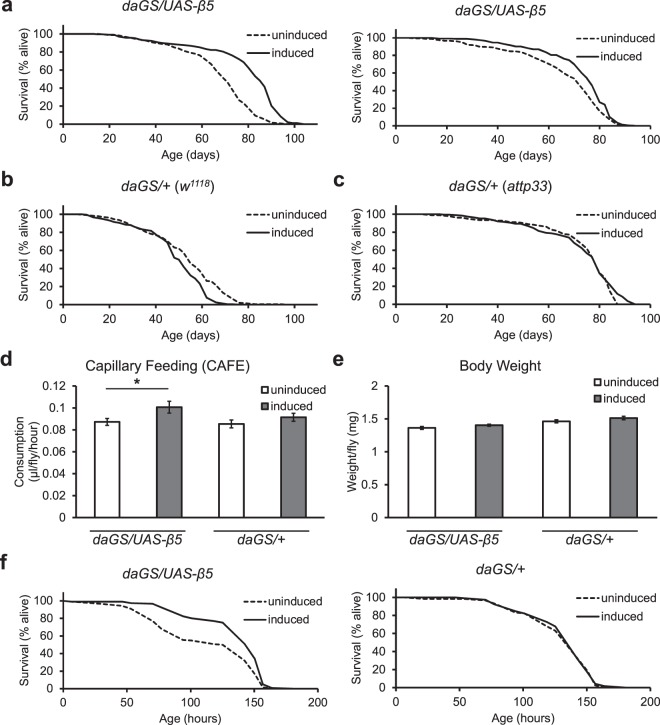
Moderate overexpression of the β5 subunit extends longevity without decreasing feeding. (**a**) β5 subunit overexpression throughout adulthood results in 10–15% increases in mean lifespan of mated females in two independent replicates (left, at least 230 flies per condition, right, at least 150 flies per condition). p < 0.001, log-rank test. Presence of mifepristone alone caused a significant decrease (b, p < 0.001, log-rank test) or no effect (c, p > 0.05, log-rank test) in survivorship of two different control flies (driver crossed to (**b**) *w*^1118^ or (**c**) *attp33* background lines). (**d**) Feeding at 13 dpe was increased in flies overexpressing β5 but not in control flies. 10 replicates, 10 flies per replicate, *p < 0.05, t-test. (**e**) Increased feeding did not result in significant differences in weight at 13 dpe (11 replicates, 10 flies per replicate, p > 0.05, t-test), but resulted in (**f**) improved wet starvation resistance (approx. 120 flies per condition, p < 0.001, log-rank test). All error bars represent standard error.

The chymotrypsin-like activity of the proteasome is the major activity for proteasomal degradation^56^ but whether boosting it, in the absence of broad transcriptional upregulation of all other subunits of the proteasome, is sufficient to improve protein homoeostasis, longevity, and stress resistance has not been investigated. Additionally, development time specific analyses to study the effects of β5 overexpression only during adulthood has not been reported. Accordingly, we set out to determine if increased β5 expression only during adulthood is sufficient to extend lifespan. We restricted our assays to female flies due to previous reports that showed greater relative decline in 26S proteasome during aging in females relative to males^32^. We found that compared to uninduced controls, flies that moderately overexpress the β5 subunit had a mean lifespan that was 10–15% longer, diverging away from the uninduced controls by approximately 50 dpe in two independent repeat experiments (Fig. 2a). Moreover, as the use of the inducing drug, mifepristone/RU486, has recently been reported to influence lifespan at high doses^57,58^, we restricted our inductions to relatively low doses and checked to ensure absence of any positive effects of dietary mifepristone with driver crosses into two different background lines, a standard laboratory *w*^1118^ line and the *attp33* line used to generate our transgenic flies (“*daGS/+* (*w*^1118^)” and “*daGS/+* (*attp33*)” in Fig. 2b,c). Even with the relatively low doses, we find that dietary mifepristone results in a significant decrease in mean lifespan of *daGS/+ *(*w*^1118^) flies (~8%) and no significant difference in lifespan of *daGS/+* (*attp33*) flies (see Supplementary Table S1). Therefore, while the inducing drug may have masked some of the benefits of β5 overexpression, it is unlikely that the positive effects we see from β5 overexpression can be attributed to the previously reported effects of high mifepristone doses.

Lifespan extensions may also result from an indirect, genetic dietary restriction, where the experimental manipulation causes an indirect decrease in feeding which induces dietary restriction on the flies. To check this possibility, we assayed multiple parameters associated with feeding and nutrition. Measurements of feeding (capillary feeding assay)^59^ showed no decrease in feeding in response to approximately 10 days of β5 induction (Fig. 2d), and there was no significant difference in weight (Fig. 2e). Instead, the feeding assay revealed a small but significant increase in feeding which may be associated with the greater survival we observed under water-only starvation conditions (Fig. 2f). Therefore, overexpression of β5 does not result in an indirect dietary restriction and likely acts through the modulation of a proteasome activity-specific pathway.

### Increased mid-life chymotrypsin-like activity does not improve acute proteotoxic stress resistance or mitochondrial markers

**Figure 3:**
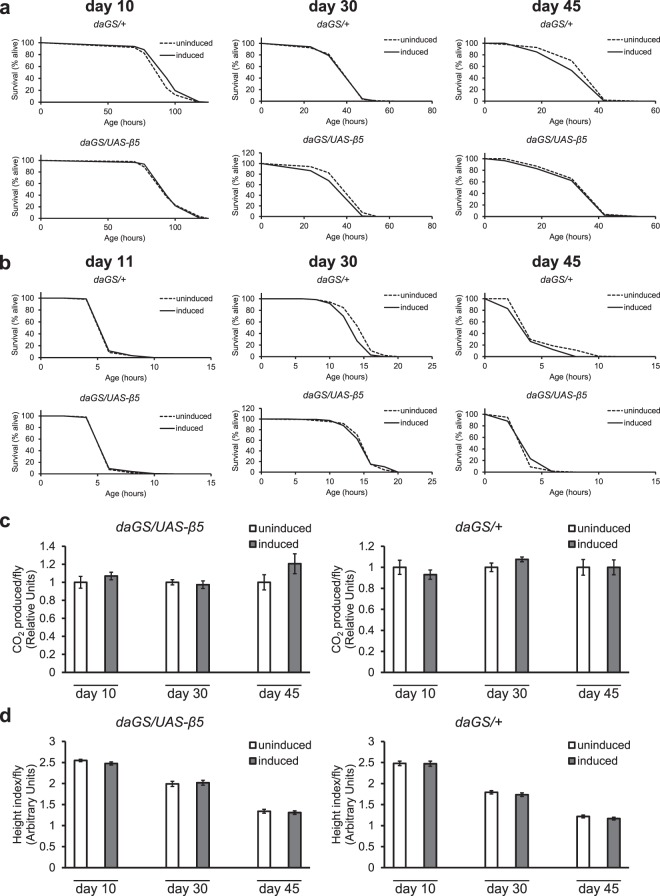
Moderate overexpression of the β5 subunit does not improve stress resistance or metabolic parameters. (**a**) Under severe hyperoxia conditions (>92% O_2_), presence of mifepristone in driver-only control flies caused a significant increase in survival at 10 dpe (~4% increase in mean, p = 0.015), no significant changes at 30 dpe (p = 0.72), and a significant decrease in survival at 45 dpe (~8% decrease in mean, p = 0.006). Under the same conditions, overexpression of the β5 subunit resulted in no significant increase in survival at any time point (no significant difference at 10 dpe, a significant decrease at 30 dpe (~8% decrease in mean, p = 0.008), and no significant difference at 45 dpe). Approx. 120 flies per condition, log-rank test. (**b**) Under elevated temperatures (37 °C), presence of mifepristone in driver-only control flies caused a decrease in survival over time (no significant difference at 11 dpe, significant decrease at 30 dpe (~7% decrease in mean, p < 0.0001) and 45 dpe (~15% decrease in mean, p = 0.0001). Under the same conditions, overexpression of the β5 subunit resulted in no significant differences in survival at any time point. Approx. 150 flies per condition, log-rank test. (**c**) Increased β5 expression during adulthood did not correlate with altered CO_2_ production (4–5 replicates, 5 flies per replicate, t-test) and (**d**) did not influence climbing rates (5 technical replicates of 5 experimental replicates, 15 flies per experimental replicate, t-test). All error bars represent standard error.

The majority of effects of increased proteasome activity are those associated with increased proteotoxic stress resistance^60^. Whether increasing only the β5 subunit of the proteasome during adulthood, without corresponding increases in transcription of every other proteasome subunit, is sufficient for the reported increases in resistance to oxidative or heat stress is unknown, however. In order to determine what effects the increased chymotrypsin-like activity has on acute proteotoxic stress resistance, we assayed survival of β5 overexpression flies under severe hyperoxia (>92% O_2_) or high temperatures (37 °C) as both are expected to reduce protein homeostasis. To determine the relationship between onset of increased chymotrypsin activity and proteotoxic stress resistance, we ran each assay at three different time points during adulthood (11, 30, and 45 dpe). We found no significant increase in survival under severely hyperoxic (Fig. 3a) or hyperthermic (Fig. 3b) conditions in the β5 overexpression flies at any time point, suggesting that a moderate increase in β5 expression and corresponding increase in chymotrypsin-like activity detected at 30 dpe are likely insufficient to significantly increase survival under our relatively harsh conditions. Moreover, this suggests that simultaneous elevation of the remaining proteasome-associated activities may be required for the reported dramatic increases in resistances to proteotoxic stresses^35–38^. An important caveat to our results is the presence of a significant effect of the inducing drug in control flies (Fig. 3a,b, see Supplementary Table S1). Driver-only control flies treated with the inducing drug revealed small but significant deterioration of stress resistance over time, which could have masked the presence of relatively small, positive effects on stress resistance in the β5 overexpression lines.

In addition to improved oxidative stress resistance, the proteasome has multiple reported functions in the regulation of mitochondrial quality and function. Increased proteostasis has been linked with improved metabolic parameters^34,61^, and increased metabolic parameters has been successfully linked with longer lifespans^62–64^. Therefore, another way in which increasing β5 expression may increase longevity is by improving metabolic parameters. To determine if increased β5 expression and chymotrypsin-like activity is sufficient to improve metabolism during aging, we assayed *in vivo* respiration (CO_2_ production, Fig. 3c) and climbing ability (Fig. 3d) in flies at 10, 30, and 45 dpe. None of the flies displayed any significant differences in respiration or climbing ability relative to their age matched controls throughout the assay period, suggesting that the increased lifespan is not a result of improved metabolism in flies that overexpress the β5 subunit.

### Increased chymotrypsin-like activity decreases protein aggregates

**Figure 4:**
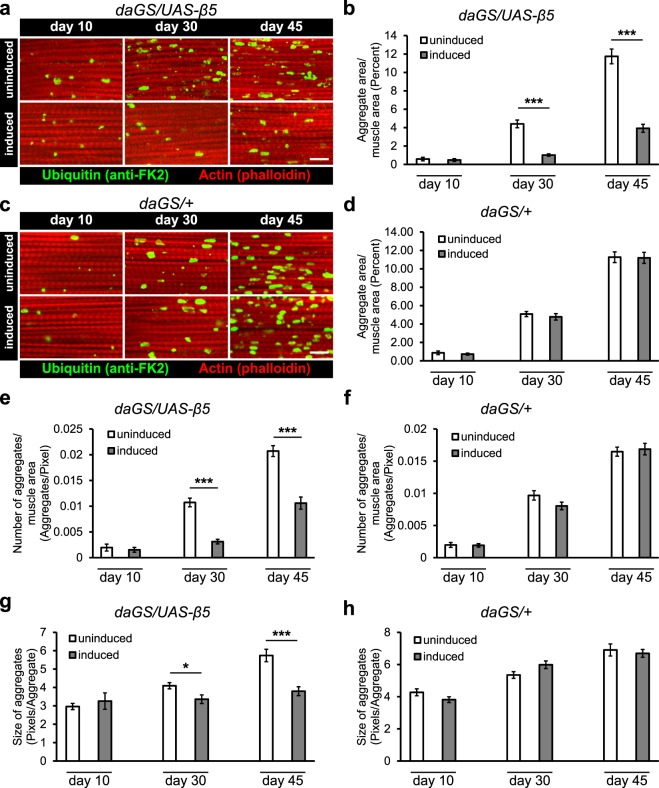
Increased chymotrypsin-like activity of the proteasome improves proteostasis during aging. (**a**) Representative images of the indirect flight muscles stained with phalloidin (red) and ubiquitin antibodies (anti-FK2, green) at three different time points (10, 30, and 45 dpe) are shown. (**a**,**b**) Overexpression of the β5 subunit results in significantly lower proportions of the tissues being labeled with anti-ubiquitin antibodies. (**c**,**d**) Drug treatment alone resulted in no significant differences at all time points. The lower proportion of anti-ubiquitin staining in flies that overexpress β5 was due to a significant reduction in the number of aggregates (**e**) and in the average size of aggregates (**g**). Both parameters were unchanged in drug control flies (**f**,**h**). At least 7 different hemi-thoraces imaged per time point. *p < 0.05, ***p < 0.001, t-test. All error bars represent standard error. Scale bar represents 10 μm.

Along with metabolic decline, loss of proteostasis is a widely recognized hallmark of aging^2^ and one that is directly impacted by the proteasome. Although overexpression of multiple proteasome subunits have been demonstrated to be sufficient to ameliorate the detrimental effects associated with the presence of protein aggregates^35–38^, whether overexpressing only the β5 subunit can be an effective therapeutic against protein aggregates has not been reported. Consequently, we assayed the presence of ubiquitinated protein aggregates in the indirect flight muscles of flies at multiple ages in flies that overexpress the β5 subunit. Despite the lack of changes in metabolic parameters, overexpression of the β5 subunit was sufficient to cause significant changes to the prevalence of these protein aggregates in our experimental flies (Fig. 4a,b) which could not be attributable to the presence of the inducing drug (Fig. 4c,d). Moreover, the reduction in total aggregate area resulted from both a significant reduction in the number (Fig. 4e,f) and average size of ubiquitin tagged protein aggregates (Fig. 4g,h) during mid-life and late-life, demonstrating that overexpression of the β5 subunit and the consequent increase in chymotrypsin-like activity of the proteasome that is observed between 10 and 45 dpe is sufficient to reduce both the number and average size of aging-associated protein aggregates at 30 and 45 dpe and that these decreases in protein aggregates are associated with longer lifespans.

## Discussion

We report that a moderate increase in expression of the β5 subunit of the proteasome throughout adulthood results in a significant boost in chymotrypsin-like activity of the proteasome and a significant increase in longevity. Increases in β5 expression and chymotrypsin-like activity did not result in flies with altered metabolism or stronger proteotoxic stress resistance, but resulted in significant reduction in the increase of ubiquitinated protein aggregates that is a hallmark of aging. Our work suggests that the β5 subunit may not be as involved in transcriptional activation of the other subunits of the proteasome in adult fruit flies compared to other models in which it has been studied, but its overexpression is nevertheless sufficient to increase chymotrypsin-like activity and increase lifespan.

Proteasome activity has been shown to decline with age^32^ and increasing proteasome function is known to provide benefits to lifespan^30,31^. Given the multiple roles that the proteasome plays, however, including roles in metabolism, cell proliferation, and cell signaling, among others^33,65^, discerning which aspects of proteasome function are limiting specifically for aging is necessary for further targeted investigations into the molecular consequences of aging. The major proteolytic activity associated with the proteasome is the chymotrypsin-like activity provided by the β5 subunit^56^, and artificial impairment of only the chymotrypsin-like activity of the proteasome in mice has been shown to be sufficient to cause multiple early aging phenotypes, including shortened lifespans, reduced body weight, altered metabolism, muscle atrophy, and accumulation of ubiquitinated peptides^40^. In contrast, in flies, additional chymotrypsin activity did not significantly influence body weight, metabolism, or motor performance, possibly pointing to the presence of redundancies in the control of these physiological parameters. In support of previous studies of the role of proteasome in aging, however, we find that boosting the chymotrypsin-like activity is sufficient to decrease both the size and number of ubiquitinated protein aggregates and to significantly extend lifespan, suggesting that the proteasome-associated chymotrypsin-like activity is limiting for these parameters.

Previous reports have shown that some subunits of the proteasome are regulatory and their overexpression results in a co-upregulation of multiple other subunits^35–38,66^. Among these, the β5 subunit in particular has been shown to be regulatory in multiple models, increasing proteasome holoenzyme activity in human cell cultures and *C. elegans*^38,66^. In contrast, we find that overexpression of the *D. melanogaster* homolog of β5 does not result in upregulation of multiple CP or RP subunits in *D. melanogaster* during an approximately 20-fold increase in β5 expression. While our results suggest that overexpression of the β5 subunit during adulthood is not sufficient to cause a concomitant increase in the transcript levels of all other proteasome subunits, previous reports have uncovered strict time-^67^ and tissue-dependent^68^ responses to proteasome induction with aged somatic tissues having poorest responses to proteasome induction by stress response pathways. While the current report focuses on adult-only, whole-organism effects of β5 overexpression, tissue- and developmental time- specific analyses of β5 overexpression may yield further insights into whether there may be subsets of tissues or developmental times during which β5 may act in a regulatory manner, if such responses are involved in the phenotypes we observe, and how tissue- and time- dependent upregulation of proteasomes by specific proteasome subunits and stress responsive pathways may be related.

Regardless of the apparent lack of compensatory expression of all other proteasome subunits, we find that expression of the β5 subunit is sufficient to cause a significant increase in the chymotrypsin-like activity associated with the proteasome. How the overexpression of one subunit might cause the elevation of the entire proteasome remains to be elucidated but a likely explanation would be that excess, unassembled subunits of the proteasome may be available and that the β5 subunit is limiting for proteasome assembly. Previous reports show that the assembly of the mature proteasome is a complex, multi-step process, during which a chaperone pro-domain of the β5 subunit play important roles in assembly, including in *trans*^69^, which suggests that unassembled β5 subunits could be key regulators of proteasome assembly. Multiple partial and intermediate assemblies of the proteasome are also known, and there is growing evidence that non-canonical CP assemblies are evolutionarily conserved^29,70^, which leaves open the possibility that an overabundance of the β5 subunit relative to the other subunits, over time, might even result in the formation of alternative complexes with different properties. An additional avenue for further work will be to examine the interplay between β5 expression, autophagy and aging.

Regardless of how the chymotrypsin-like activity can be augmented without concomitant increase of the expression of the other proteasome subunits, which proteins are degraded in a manner limited by the chymotrypsin-like activity of the proteasome may shed light on the proteotoxic elements that build up during the aging process which may be critical to longevity. Overexpression of the β5 subunit in the experimentally versatile and powerful *D. melanogaster* provides means for elucidating further molecular details on the proteasome function and its specific interactions that influence the aging process.

## Supplementary information

Dataset 1

## Data Availability

The data collected and analyzed for the current study are available from the corresponding author upon request.
