# Supplementary material for: Proteasome β5 subunit overexpression improves proteostasis during aging and extends lifespan in Drosophila melanogaster
Source: Sci Rep. 2019 Feb 28;9:3170. doi: 10.1038/s41598-019-39508-4 (PMC6395709; doi:10.1038/s41598-019-39508-4)
Supplement: Supplementary file 1 — Dataset 1 [file 41598_2019_39508_MOESM1_ESM.docx]

Supplementary Material for ‘Proteasome β5 subunit overexpression improves proteostasis during aging and extends lifespan in *Drosophila melanogaster*’

Nga N. Nguyen, Anil Rana, Camille Goldman, Rhiannon Moore, Justin Tai, Yongchan Hong, Jingyi Shen, David W. Walker, Jae H. Hur


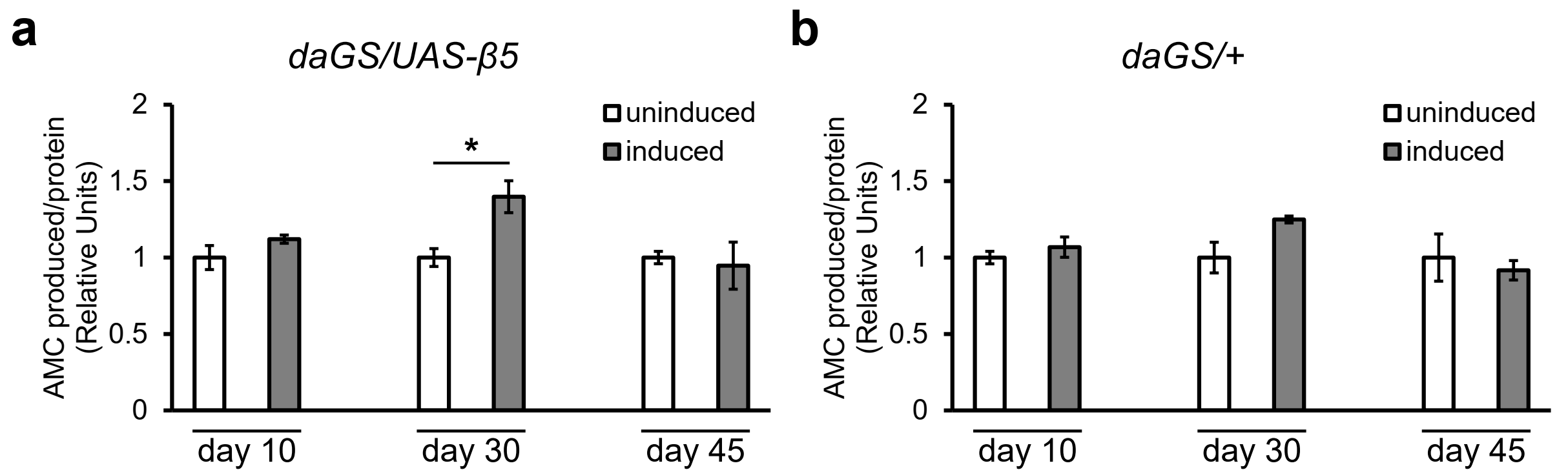


**Figure S1. β5 subunit overexpression during adulthood results in increased 26S/30S chymotrypsin-like activity.** (a) Induction of β5 subunit overexpression during adulthood in mated female flies results in a significant elevation in 26S/30S proteasome specific chymotrypsin-like activity by 30 dpe, but no significant difference at 10 and 45 dpe. (b) The presence of inducing drug in control flies did not result in a significant change to chymotrypsin-like activity at any time point. 5 replicates, 5 heads and thoraces per replicate. * p < 0.05, t-test. All error bars represent standard error.

Table S1. Mean, standard error, statistical test, and p values of all presented data.

| Figure # | Panel | Category | Condition | Mean | Standard Error (±) | Statistical Test | p Value |
| --- | --- | --- | --- | --- | --- | --- | --- |
|  |  |  |  |  |  |  |  |
| 1 | a | beta5 | daGS/+ | 1 | 0.04317 | t-test (heteroscedastic) | 0.00033 |
|  |  | beta5 | daGS/UAS-β5 | 18.4915 | 1.32471 |  |  |
|  |  | beta1 | daGS/+ | 1 | 0.06072 | t-test (homoscedastic) | 0.39727 |
|  |  | beta1 | daGS/UAS-β5 | 1.04582 | 0.115 |  |  |
|  |  | beta2 | daGS/+ | 1 | 0.0884 | t-test (homoscedastic) | 0.02971 |
|  |  | beta2 | daGS/UAS-β5 | 1.19805 | 0.10024 |  |  |
|  |  | alpha2 | daGS/+ | 1 | 0.04723 | t-test (homoscedastic) | 0.63386 |
|  |  | alpha2 | daGS/UAS-β5 | 0.96862 | 0.06274 |  |  |
|  |  | alpha3 | daGS/+ | 1 | 0.04274 | t-test (homoscedastic) | 0.21398 |
|  |  | alpha3 | daGS/UAS-β5 | 0.89179 | 0.05349 |  |  |
|  |  | Rpn10 | daGS/+ | 1 | 0.10748 | t-test (homoscedastic) | 0.63703 |
|  |  | Rpn10 | daGS/UAS-β5 | 1.05214 | 0.17024 |  |  |
|  |  | Rpn13 | daGS/+ | 1 | 0.02719 | t-test (homoscedastic) | 0.13934 |
|  |  | Rpn13 | daGS/UAS-β5 | 1.11936 | 0.11768 |  |  |
|  |  | Rpt2 | daGS/+ | 1 | 0.0225 | t-test (heteroscedastic) | 0.8837 |
|  |  | Rpt2 | daGS/UAS-β5 | 0.95775 | 0.10791 |  |  |
|  |  | Rpt3 | daGS/+ | 1 | 0.09393 | t-test (homoscedastic) | 0.21442 |
|  |  | Rpt3 | daGS/UAS-β5 | 1.05392 | 0.07343 |  |  |
|  |  | Rpn6 | daGS/+ | 1 | 0.03329 | t-test (homoscedastic) | 0.27602 |
|  |  | Rpn6 | daGS/UAS-β5 | 1.0412 | 0.07624 |  |  |
|  |  | Rpn11 | daGS/+ | 1 | 0.04273 | t-test (homoscedastic) | 0.05504 |
|  |  | Rpn11 | daGS/UAS-β5 | 1.03173 | 0.0449 |  |  |
|  |  |  |  |  |  |  |  |
|  | b | beta5 | daGS/+ | 1 | 0.05197 | t-test (heteroscedastic) | 0.00018 |
|  |  | beta5 | daGS/UAS-β5 | 17.9048 | 1.26128 |  |  |
|  |  | beta1 | daGS/+ | 1 | 0.08293 | t-test (homoscedastic) | 0.98121 |
|  |  | beta1 | daGS/UAS-β5 | 1.00308 | 0.09603 |  |  |
|  |  |  |  |  |  |  |  |
|  |  | beta2 | daGS/+ | 1 | 0.06547 | t-test (homoscedastic) | 0.13563 |
|  |  | beta2 | daGS/UAS-β5 | 1.16305 | 0.07328 |  |  |
|  |  | alpha2 | daGS/+ | 1 | 0.05939 | t-test (homoscedastic) | 0.40171 |
|  |  | alpha2 | daGS/UAS-β5 | 0.93379 | 0.04542 |  |  |
|  |  | alpha3 | daGS/+ | 1 | 0.04771 | t-test (homoscedastic) | 0.05489 |
|  |  | alpha3 | daGS/UAS-β5 | 0.86163 | 0.03897 |  |  |
|  |  | Rpn10 | daGS/+ | 1 | 0.13121 | t-test (homoscedastic) | 0.99275 |
|  |  | Rpn10 | daGS/UAS-β5 | 0.99816 | 0.14529 |  |  |
|  |  | Rpn13 | daGS/+ | 1 | 0.05601 | t-test (homoscedastic) | 0.52812 |
|  |  | Rpn13 | daGS/UAS-β5 | 1.07522 | 0.09937 |  |  |
|  |  | Rpt2 | daGS/+ | 1 | 0.02846 | t-test (homoscedastic) | 0.41284 |
|  |  | Rpt2 | daGS/UAS-β5 | 0.92078 | 0.08718 |  |  |
|  |  | Rpt3 | daGS/+ | 1 | 0.0877 | t-test (homoscedastic) | 0.84391 |
|  |  | Rpt3 | daGS/UAS-β5 | 1.02043 | 0.04896 |  |  |
|  |  | Rpn6 | daGS/+ | 1 | 0.06064 | t-test (homoscedastic) | 0.96521 |
|  |  | Rpn6 | daGS/UAS-β5 | 1.0042 | 0.07089 |  |  |
|  |  | Rpn11 | daGS/+ | 1 | 0.04042 | t-test (homoscedastic) | 0.98553 |
|  |  | Rpn11 | daGS/UAS-β5 | 0.99907 | 0.02856 |  |  |
|  |  |  |  |  |  |  |  |
|  | c | beta5 | daGS/+ | 1 | 0.03548 | t-test (heteroscedastic) | 0.00042 |
|  |  | beta5 | daGS/UAS-β5 | 20.1245 | 1.77635 |  |  |
|  |  | beta1 | daGS/+ | 1 | 0.03075 | t-test (homoscedastic) | 0.23497 |
|  |  | beta1 | daGS/UAS-β5 | 1.12676 | 0.09379 |  |  |
|  |  | beta2 | daGS/+ | 1 | 0.09902 | t-test (homoscedastic) | 0.0432 |
|  |  | beta2 | daGS/UAS-β5 | 1.27985 | 0.06161 |  |  |
|  |  | alpha2 | daGS/+ | 1 | 0.08196 | t-test (homoscedastic) | 0.73176 |
|  |  | alpha2 | daGS/UAS-β5 | 1.03231 | 0.03958 |  |  |
|  |  | alpha3 | daGS/+ | 1 | 0.07272 | t-test (homoscedastic) | 0.54967 |
|  |  | alpha3 | daGS/UAS-β5 | 0.9515 | 0.02728 |  |  |
|  |  | Rpn10 | daGS/+ | 1 | 0.08054 | t-test (homoscedastic) | 0.46559 |
|  |  | Rpn10 | daGS/UAS-β5 | 1.1233 | 0.13933 |  |  |
|  |  |  |  |  |  |  |  |
|  |  | Rpn13 | daGS/+ | 1 | 0.02908 | t-test (heteroscedastic) | 0.0967 |
|  |  | Rpn13 | daGS/UAS-β5 | 1.19725 | 0.09122 |  |  |
|  |  | Rpt2 | daGS/+ | 1 | 0.04722 | t-test (homoscedastic) | 0.86572 |
|  |  | Rpt2 | daGS/UAS-β5 | 1.01568 | 0.07639 |  |  |
|  |  | Rpt3 | daGS/+ | 1 | 0.06302 | t-test (homoscedastic) | 0.08412 |
|  |  | Rpt3 | daGS/UAS-β5 | 1.14077 | 0.03355 |  |  |
|  |  | Rpn6 | daGS/+ | 1 | 0.05167 | t-test (homoscedastic) | 0.23482 |
|  |  | Rpn6 | daGS/UAS-β5 | 1.12035 | 0.07814 |  |  |
|  |  | Rpn11 | daGS/+ | 1 | 0.05775 | t-test (homoscedastic) | 0.13916 |
|  |  | Rpn11 | daGS/UAS-β5 | 1.11032 | 0.03432 |  |  |
|  |  |  |  |  |  |  |  |
|  | d | beta5 | daGS/+ | 1 | 0.04317 | t-test (heteroscedastic) | 0.00019 |
|  |  | beta5 | daGS/UAS-β5 | 18.4915 | 1.32471 |  |  |
|  |  | beta1 | daGS/+ | 1 | 0.06072 | t-test (homoscedastic) | 0.73367 |
|  |  | beta1 | daGS/UAS-β5 | 1.04582 | 0.115 |  |  |
|  |  | beta2 | daGS/+ | 1 | 0.0884 | t-test (homoscedastic) | 0.17667 |
|  |  | beta2 | daGS/UAS-β5 | 1.19805 | 0.10024 |  |  |
|  |  | alpha2 | daGS/+ | 1 | 0.04723 | t-test (homoscedastic) | 0.69995 |
|  |  | alpha2 | daGS/UAS-β5 | 0.96862 | 0.06274 |  |  |
|  |  | alpha3 | daGS/+ | 1 | 0.04274 | t-test (homoscedastic) | 0.15266 |
|  |  | alpha3 | daGS/UAS-β5 | 0.89179 | 0.05349 |  |  |
|  |  | Rpn10 | daGS/+ | 1 | 0.10748 | t-test (homoscedastic) | 0.80219 |
|  |  | Rpn10 | daGS/UAS-β5 | 1.05214 | 0.17024 |  |  |
|  |  | Rpn13 | daGS/+ | 1 | 0.02719 | t-test (heteroscedastic) | 0.37392 |
|  |  | Rpn13 | daGS/UAS-β5 | 1.11936 | 0.11768 |  |  |
|  |  | Rpt2 | daGS/+ | 1 | 0.0225 | t-test (heteroscedastic) | 0.71954 |
|  |  | Rpt2 | daGS/UAS-β5 | 0.95775 | 0.10791 |  |  |
|  |  | Rpt3 | daGS/+ | 1 | 0.09393 | t-test (homoscedastic) | 0.6631 |
|  |  | Rpt3 | daGS/UAS-β5 | 1.05392 | 0.07343 |  |  |
|  |  | Rpn6 | daGS/+ | 1 | 0.03329 | t-test (homoscedastic) | 0.63373 |
|  |  | Rpn6 | daGS/UAS-β5 | 1.0412 | 0.07624 |  |  |
|  |  |  |  |  |  |  |  |
|  |  | Rpn11 | daGS/+ | 1 | 0.04273 | t-test (homoscedastic) | 0.62256 |
|  |  | Rpn11 | daGS/UAS-β5 | 1.03173 | 0.0449 |  |  |
|  |  |  |  |  |  |  |  |
|  | e | daGS/UAS-β5 | uninduced | 0.98462 | 0.16469 | t-test (heteroscedastic) | 0.03658 |
|  |  | daGS/UAS-β5 | induced | 2.01605 | 0.39874 |  |  |
|  |  | daGS/+ | uninduced | 0.72807 | 0.08137 | t-test (homoscedastic) | 0.89072 |
|  |  | daGS/+ | induced | 0.71202 | 0.08124 |  |  |
|  |  |  |  |  |  |  |  |
|  | f | daGS/UAS-β5 | uninduced | 1 | 0.05851 | t-test (homoscedastic) | 0.01594 |
|  |  | daGS/UAS-β5 | induced | 1.39794 | 0.10449 |  |  |
|  |  | daGS/+ | uninduced | 1 | 0.10054 | t-test (heteroscedastic) | 0.06706 |
|  |  | daGS/+ | induced | 1.24922 | 0.02246 |  |  |
|  |  |  |  |  |  |  |  |
|  |  |  |  |  |  |  |  |
| 2 | a | daGS/UAS-β5 (repeat 1, left) | uninduced | 67.2573 |  | log-rank test | < 0.0001 |
|  |  | daGS/UAS-β5 (repeat 1, left) | induced | 78.7669 |  |  |  |
|  |  | daGS/UAS-β5 (repeat 2, right) | uninduced | 66.0641 |  | log-rank test | 0.001 |
|  |  | daGS/UAS-β5 (repeat 2, right) | induced | 72.6144 |  |  |  |
|  |  |  |  |  |  |  |  |
|  | b | daGS/+ (w1118) | uninduced | 52.9959 |  | log-rank test | < 0.0001 |
|  |  | daGS/+ (w1118) | induced | 48.617 |  |  |  |
|  |  |  |  |  |  |  |  |
|  | c | daGS/+ (attp33) | uninduced | 73.1492 |  | log-rank test | 0.1279 |
|  |  | daGS/+ (attp33) | induced | 73.0635 |  |  |  |
|  |  |  |  |  |  |  |  |
|  | d | daGS/UAS-β5 | uninduced | 0.08721 | 0.00315 | t-test (homoscedastic) | 0.04547 |
|  |  | daGS/UAS-β5 | induced | 0.10065 | 0.0054 |  |  |
|  |  | daGS/+ | uninduced | 0.0854 | 0.0036 | t-test (homoscedastic) | 0.24912 |
|  |  | daGS/+ | induced | 0.0915 | 0.00365 |  |  |
|  |  |  |  |  |  |  |  |
|  | e | daGS/UAS-β5 | uninduced | 1.36273 | 0.02195 | t-test (homoscedastic) | 0.15359 |
|  |  | daGS/UAS-β5 | induced | 1.40364 | 0.01669 |  |  |
|  |  | daGS/+ | uninduced | 1.46273 | 0.02367 | t-test (homoscedastic) | 0.17644 |
|  |  | daGS/+ | induced | 1.51273 | 0.0267 |  |  |
|  |  |  |  |  |  |  |  |
|  | f | daGS/UAS-β5 | uninduced | 113.46 |  | log-rank test | < 0.0001 |
|  |  | daGS/UAS-β5 | induced | 136.319 |  |  |  |
|  |  | daGS/+ | uninduced | 131.408 |  | log-rank test | 0.6752 |
|  |  | daGS/+ | induced | 133.469 |  |  |  |
|  |  |  |  |  |  |  |  |
|  |  |  |  |  |  |  |  |
| 3 | a | 10 dpe (daGS/+) | uninduced | 93.8246 |  | log-rank test | 0.015 |
|  |  | 10 dpe (daGS/+) | induced | 97.3312 |  |  |  |
|  |  | 30 dpe (daGS/+) | uninduced | 43.9958 |  | log-rank test | 0.7243 |
|  |  | 30 dpe (daGS/+) | induced | 43.7353 |  |  |  |
|  |  | 45 dpe (daGS/+) | uninduced | 37.6818 |  | log-rank test | 0.006 |
|  |  | 45 dpe (daGS/+) | induced | 34.7575 |  |  |  |
|  |  | 10 dpe (daGS/UAS-β5) | uninduced | 98.321 |  | log-rank test | 0.9719 |
|  |  | 10 dpe (daGS/UAS-β5) | induced | 98.7266 |  |  |  |
|  |  | 30 dpe (daGS/UAS-β5) | uninduced | 44.5229 |  | log-rank test | 0.0008 |
|  |  | 30 dpe (daGS/UAS-β5) | induced | 41.0847 |  |  |  |
|  |  | 45 dpe (daGS/UAS-β5) | uninduced | 36.9352 |  | log-rank test | 0.4179 |
|  |  | 45 dpe (daGS/UAS-β5) | induced | 35.4612 |  |  |  |
|  |  |  |  |  |  |  |  |
|  | b | 11 dpe (daGS/+) | uninduced | 6.2 |  | log-rank test | 0.5668 |
|  |  | 11 dpe (daGS/+) | induced | 6.26667 |  |  |  |
|  |  | 30 dpe (daGS/+) | uninduced | 14.869 |  | log-rank test | < 0.0001 |
|  |  | 30 dpe (daGS/+) | induced | 13.8143 |  |  |  |
|  |  | 45 dpe (daGS/+) | uninduced | 5.2 |  | log-rank test | 0.0001 |
|  |  | 45 dpe (daGS/+) | induced | 4.42553 |  |  |  |
|  |  | 11 dpe (daGS/UAS-β5) | uninduced | 6.14667 |  | log-rank test | 0.44 |
|  |  | 11 dpe (daGS/UAS-β5) | induced | 6.22667 |  |  |  |
|  |  | 30 dpe (daGS/UAS-β5) | uninduced | 15.4583 |  | log-rank test | 0.8734 |
|  |  | 30 dpe (daGS/UAS-β5) | induced | 15.4324 |  |  |  |
|  |  | 45 dpe (daGS/UAS-β5) | uninduced | 4.10606 |  | log-rank test | 0.2635 |
|  |  | 45 dpe (daGS/UAS-β5) | induced | 4.22388 |  |  |  |
|  |  |  |  |  |  |  |  |
|  | c | 10 dpe (daGS/UAS-β5) | uninduced | 1 | 0.06536 | t-test (homoscedastic) | 0.39517 |
|  |  | 10 dpe (daGS/UAS-β5) | induced | 1.06975 | 0.04189 |  |  |
|  |  | 30 dpe (daGS/UAS-β5) | uninduced | 1 | 0.02869 | t-test (homoscedastic) | 0.63824 |
|  |  | 30 dpe (daGS/UAS-β5) | induced | 0.97333 | 0.0424 |  |  |
|  |  | 45 dpe (daGS/UAS-β5) | uninduced | 1 | 0.08455 | t-test (homoscedastic) | 0.17644 |
|  |  | 45 dpe (daGS/UAS-β5) | induced | 1.20635 | 0.11054 |  |  |
|  |  | 10 dpe (daGS/+) | uninduced | 1 | 0.06739 | t-test (homoscedastic) | 0.41269 |
|  |  | 10 dpe (daGS/+) | induced | 0.93035 | 0.04422 |  |  |
|  |  | 30 dpe (daGS/+) | uninduced | 1 | 0.04074 | t-test (homoscedastic) | 0.14254 |
|  |  | 30 dpe (daGS/+) | induced | 1.07563 | 0.02243 |  |  |
|  |  | 45 dpe (daGS/+) | uninduced | 1 | 0.07476 | t-test (homoscedastic) | 1 |
|  |  | 45 dpe (daGS/+) | induced | 1 | 0.07067 |  |  |
|  |  |  |  |  |  |  |  |
|  | d | 10 dpe (daGS/UAS-β5) | uninduced | 2.54743 | 0.029 | t-test (homoscedastic) | 0.17496 |
|  |  | 10 dpe (daGS/UAS-β5) | induced | 2.47619 | 0.03807 |  |  |
|  |  | 30 dpe (daGS/UAS-β5) | uninduced | 1.99067 | 0.06168 | t-test (homoscedastic) | 0.75061 |
|  |  | 30 dpe (daGS/UAS-β5) | induced | 2.01848 | 0.0578 |  |  |
|  |  | 45 dpe (daGS/UAS-β5) | uninduced | 1.34019 | 0.0461 | t-test (homoscedastic) | 0.63739 |
|  |  | 45 dpe (daGS/UAS-β5) | induced | 1.30952 | 0.04235 |  |  |
|  |  | 10 dpe (daGS/+) | uninduced | 2.48019 | 0.05388 | t-test (homoscedastic) | 0.92446 |
|  |  | 10 dpe (daGS/+) | induced | 2.47219 | 0.06149 |  |  |
|  |  | 30 dpe (daGS/+) | uninduced | 1.79162 | 0.03983 | t-test (homoscedastic) | 0.37042 |
|  |  | 30 dpe (daGS/+) | induced | 1.73581 | 0.04327 |  |  |
|  |  | 45 dpe (daGS/+) | uninduced | 1.2181 | 0.0335 | t-test (homoscedastic) | 0.30872 |
|  |  | 45 dpe (daGS/+) | induced | 1.16724 | 0.03266 |  |  |
|  |  |  |  |  |  |  |  |
|  |  |  |  |  |  |  |  |
| 4 | b | 10 dpe (daGS/UAS-β5) | uninduced | 0.57987 | 0.1893 | t-test (homoscedastic) | 0.68116 |
|  |  | 10 dpe (daGS/UAS-β5) | induced | 0.4774 | 0.15297 |  |  |
|  |  | 30 dpe (daGS/UAS-β5) | uninduced | 4.40581 | 0.41768 | t-test (heteroscedastic) | 5.5E-06 |
|  |  | 30 dpe (daGS/UAS-β5) | induced | 1.01599 | 0.12796 |  |  |
|  |  | 45 dpe (daGS/UAS-β5) | uninduced | 11.7398 | 0.80221 | t-test (heteroscedastic) | 1.6E-09 |
|  |  | 45 dpe (daGS/UAS-β5) | induced | 3.92505 | 0.43375 |  |  |
|  |  |  |  |  |  |  |  |
|  | d | 10 dpe (daGS/+) | uninduced | 0.86886 | 0.20219 | t-test (homoscedastic) | 0.52997 |
|  |  | 10 dpe (daGS/+) | induced | 0.7292 | 0.11238 |  |  |
|  |  | 30 dpe (daGS/+) | uninduced | 5.07932 | 0.27978 | t-test (homoscedastic) | 0.51655 |
|  |  | 30 dpe (daGS/+) | induced | 4.7788 | 0.35068 |  |  |
|  |  | 45 dpe (daGS/+) | uninduced | 11.2596 | 0.58311 | t-test (homoscedastic) | 0.93612 |
|  |  | 45 dpe (daGS/+) | induced | 11.1913 | 0.59855 |  |  |
|  |  |  |  |  |  |  |  |
|  | e | 10 dpe (daGS/UAS-β5) | uninduced | 0.00196 | 0.00067 | t-test (homoscedastic) | 0.60712 |
|  |  | 10 dpe (daGS/UAS-β5) | induced | 0.00152 | 0.00046 |  |  |
|  |  | 30 dpe (daGS/UAS-β5) | uninduced | 0.01072 | 0.00084 | t-test (homoscedastic) | 5.9E-08 |
|  |  | 30 dpe (daGS/UAS-β5) | induced | 0.00312 | 0.00045 |  |  |
|  |  | 45 dpe (daGS/UAS-β5) | uninduced | 0.02071 | 0.00105 | t-test (homoscedastic) | 3.1E-07 |
|  |  | 45 dpe (daGS/UAS-β5) | induced | 0.0106 | 0.00118 |  |  |
|  |  |  |  |  |  |  |  |
|  | f | 10 dpe (daGS/+) | uninduced | 0.00197 | 0.00038 | t-test (homoscedastic) | 0.90257 |
|  |  | 10 dpe (daGS/+) | induced | 0.00191 | 0.00027 |  |  |
|  |  | 30 dpe (daGS/+) | uninduced | 0.00968 | 0.00073 | t-test (homoscedastic) | 0.09507 |
|  |  | 30 dpe (daGS/+) | induced | 0.00804 | 0.00059 |  |  |
|  |  | 45 dpe (daGS/+) | uninduced | 0.01648 | 0.0007 | t-test (homoscedastic) | 0.74331 |
|  |  | 45 dpe (daGS/+) | induced | 0.01687 | 0.0009 |  |  |
|  |  |  |  |  |  |  |  |
|  | g | 10 dpe (daGS/UAS-β5) | uninduced | 2.96535 | 0.16883 | t-test (homoscedastic) | 0.52427 |
|  |  | 10 dpe (daGS/UAS-β5) | induced | 3.26062 | 0.44594 |  |  |
|  |  | 30 dpe (daGS/UAS-β5) | uninduced | 4.09585 | 0.1685 | t-test (homoscedastic) | 0.02121 |
|  |  | 30 dpe (daGS/UAS-β5) | induced | 3.36213 | 0.23578 |  |  |
|  |  | 45 dpe (daGS/UAS-β5) | uninduced | 5.74217 | 0.33875 | t-test (homoscedastic) | 0.00013 |
|  |  | 45 dpe (daGS/UAS-β5) | induced | 3.79818 | 0.24183 |  |  |
|  |  |  |  |  |  |  |  |
|  | h | 10 dpe (daGS/+) | uninduced | 4.27061 | 0.21638 | t-test (homoscedastic) | 0.11516 |
|  |  | 10 dpe (daGS/+) | induced | 3.81471 | 0.17903 |  |  |
|  |  | 30 dpe (daGS/+) | uninduced | 5.3469 | 0.20812 | t-test (homoscedastic) | 0.059 |
|  |  | 30 dpe (daGS/+) | induced | 5.98424 | 0.2359 |  |  |
|  |  | 45 dpe (daGS/+) | uninduced | 6.90419 | 0.37503 | t-test (homoscedastic) | 0.62902 |
|  |  | 45 dpe (daGS/+) | induced | 6.69136 | 0.24321 |  |  |
|  |  |  |  |  |  |  |  |
|  |  |  |  |  |  |  |  |
| S1 | a | 10 dpe (daGS/UAS-β5) | uninduced | 1 | 0.07856 | t-test (homoscedastic) | 0.23264 |
|  |  | 10 dpe (daGS/UAS-β5) | induced | 1.12042 | 0.02691 |  |  |
|  |  | 30 dpe (daGS/UAS-β5) | uninduced | 1 | 0.05851 | t-test (homoscedastic) | 0.01594 |
|  |  | 30 dpe (daGS/UAS-β5) | induced | 1.39794 | 0.10449 |  |  |
|  |  | 45 dpe (daGS/UAS-β5) | uninduced | 1 | 0.04051 | t-test (heteroscedastic) | 0.78187 |
|  |  | 45 dpe (daGS/UAS-β5) | induced | 0.94692 | 0.15401 |  |  |
|  |  |  |  |  |  |  |  |
|  | b | 10 dpe (daGS/+) | uninduced | 1 | 0.04097 | t-test (homoscedastic) | 0.40747 |
|  |  | 10 dpe (daGS/+) | induced | 1.0683 | 0.06653 |  |  |
|  |  | 30 dpe (daGS/+) | uninduced | 1 | 0.10054 | t-test (heteroscedastic) | 0.06706 |
|  |  | 30 dpe (daGS/+) | induced | 1.24922 | 0.02246 |  |  |
|  |  | 45 dpe (daGS/+) | uninduced | 1 | 0.15436 | t-test (homoscedastic) | 0.63182 |
|  |  | 45 dpe (daGS/+) | induced | 0.91685 | 0.06356 |  |  |
|  |  |  |  |  |  |  |  |
